# Dynamic regulation of mRNA decay during neural development

**DOI:** 10.1186/s13064-015-0038-6

**Published:** 2015-04-21

**Authors:** Dana A Burow, Maxine C Umeh-Garcia, Marie B True, Crystal D Bakhaj, David H Ardell, Michael D Cleary

**Affiliations:** Quantitative and Systems Biology Graduate Program, University of California, 5200 N. Lake Rd, Merced, CA USA

**Keywords:** Gene expression, mRNA decay, Transcriptome, Neurogenesis, Pumilio

## Abstract

**Background:**

Gene expression patterns are determined by rates of mRNA transcription and decay. While transcription is known to regulate many developmental processes, the role of mRNA decay is less extensively defined. A critical step toward defining the role of mRNA decay in neural development is to measure genome-wide mRNA decay rates in neural tissue. Such information should reveal the degree to which mRNA decay contributes to differential gene expression and provide a foundation for identifying regulatory mechanisms that affect neural mRNA decay.

**Results:**

We developed a technique that allows genome-wide mRNA decay measurements in intact *Drosophila* embryos, across all tissues and specifically in the nervous system. Our approach revealed neural-specific decay kinetics, including stabilization of transcripts encoding regulators of axonogenesis and destabilization of transcripts encoding ribosomal proteins and histones. We also identified correlations between mRNA stability and physiologic properties of mRNAs; mRNAs that are predicted to be translated within axon growth cones or dendrites have long half-lives while mRNAs encoding transcription factors that regulate neurogenesis have short half-lives. A search for candidate *cis*-regulatory elements identified enrichment of the Pumilio recognition element (PRE) in mRNAs encoding regulators of neurogenesis. We found that decreased expression of the RNA-binding protein Pumilio stabilized predicted neural mRNA targets and that a PRE is necessary to trigger reporter-transcript decay in the nervous system.

**Conclusions:**

We found that differential mRNA decay contributes to the relative abundance of transcripts involved in cell-fate decisions, axonogenesis, and other critical events during *Drosophila* neural development. Neural-specific decay kinetics and the functional specificity of mRNA decay suggest the existence of a dynamic neurodevelopmental mRNA decay network. We found that Pumilio is one component of this network, revealing a novel function for this RNA-binding protein.

**Electronic supplementary material:**

The online version of this article (doi:10.1186/s13064-015-0038-6) contains supplementary material, which is available to authorized users.

## Background

Transcription regulates neural developmental processes ranging from cell-fate specification [1] to synapse formation [2]. However, differential transcription alone is an ineffective method of regulating gene expression; mathematical models demonstrate that mRNA decay is essential for precise temporal and spatial control of mRNA abundance, particularly during developmental transitions [3]. Multiple mechanisms determine mRNA half-life [4]. One common mechanism involves the recognition of *cis*-elements by RNA-binding proteins (RBPs). RBP-mRNA interactions can activate or inhibit mRNA decay by affecting the recruitment or activity of RNA degradation complexes. Additional mRNA decay mechanisms include targeting by microRNAs and the nonsense-mediated decay (NMD) pathway. The stability of an individual transcript may differ depending on cell type or the activation of signaling pathways. These mechanisms establish mRNA decay networks in which mRNA stability is genetically programmed, tunable, and tightly regulated.

Regulation of mRNA decay appears to be particularly important for nervous system development [5]. This is partly due to the unique architecture of neurons; mRNAs exit the nucleus far from axon growth cones and dendrites where some transcripts are translated. The local concentrations of many mRNAs involved in axon pathfinding and synapse function are controlled *via* mRNA decay. For example, growth cone-localized *Robo3.2* mRNA is degraded by the NMD pathway when axons encounter the spinal cord floor plate [6]. This compartmentalized degradation of *Robo3.2* is necessary for the proper decussation of neurons in the spinal cord. Regulation of mRNA decay is also important for the proper proliferation and differentiation of neural progenitors. Mouse neural progenitors lacking the RBP HuD have increased rates of self-renewal [7], and a circuitry involving the NMD pathway and neural miRNAs controls the balance between stem-cell proliferation and neural differentiation [8]. A role for mRNA decay in regulating cell-fate specification has been identified for the *glial cells missing* (*gcm*) gene in *Drosophila* [9]. Expression of a *gcm* transgene lacking a destabilizing *cis*-element causes neural progenitors to produce excess glia. Numerous examples of microRNAs, nonsense-mediated decay, and RBPs regulating the decay of target transcripts during neural development have been described, but the degree to which differential mRNA decay globally affects mRNA abundance during *in vivo* neurogenesis is not completely defined.

The degradation of maternally deposited mRNAs in early *Drosophila* embryos has provided valuable information about the role of mRNA decay in animal development [10]. However, analysis of zygotic mRNA decay during later stages of *Drosophila* development, specifically in the nervous system, presents several technical challenges. First, it requires a method to measure mRNA decay that does not interfere with gene expression or development. Traditional approaches for measuring mRNA decay rely on transcription inhibition (using drugs or temperature-sensitive mutations that inhibit RNA polymerase II) and may have unwanted side effects [11]. Second, it requires a method to measure neural-specific mRNA decay in intact embryos. Many genes with neural development functions are expressed in multiple tissues, and the same transcript may have different half-lives in neural *versus* non-neural cells. Whole embryo mRNA decay measurements will therefore represent the aggregate half-life of an mRNA across multiple tissues.

A potential solution to the above mentioned challenges is to use a pulse-chase approach to ‘tag’ nascent mRNAs in specific cell types then follow the decay of tagged mRNAs over time. Tissue-specific expression of the *Toxoplasma gondii* uracil phosphoribosyltransferase (T.g.UPRT) enzyme in *Drosophila* allows tagging of nascent mRNAs with 4-thiouracil and subsequent purification of the tagged mRNA (a technique known as TU-tagging) [12]. Variations of TU-tagging, in which pulse-labeling is followed by a “chase” in media lacking tagged uracil, have been used to obtain genome-wide mRNA decay measurements in yeast [13] and mammalian cell lines [14]. Here we combined TU-tagging with a pulse-chase approach to obtain genome-wide measurements of mRNA decay across all tissues of *Drosophila* embryos and genome-wide measurements of mRNA decay specifically in the nervous system. This approach identified key components of a neural development mRNA decay network, including the differential decay of mRNAs within distinct functional classes and the role of the RBP Pumilio in regulating neural mRNA decay.

## Results

### TU-decay allows mRNA decay measurements in intact *Drosophila* embryos

To measure zygotic mRNA decay in *Drosophila* embryos, we developed a pulse-chase approach termed ‘TU-decay’. We first used the nucleoside 4-thiouridine (4sUd) to tag mRNAs in all embryonic tissues (independent of *T.g.UPRT*). Stage 12 to 15 embryos were incubated in 1 mM 4sUd during a 1-hour ‘pulse’ then transferred to ‘chase’ media containing 10 mM uridine. 4sUd-RNA (TU-RNA) was purified after the 1-hour pulse, a 1-hour chase, and a 3-hour chase. A potential limitation of this approach is continued incorporation of 4sUd during the chase, either due to incomplete saturation with uridine or recycling of 4sUd from degraded transcripts. 4sUd incorporation during the chase could result in artificially long half-life calculations. To test for such an effect, we performed parallel experiments in which the transcription inhibitor actinomycin D (actD) was included in the chase media. Efficient transcription arrest upon actD exposure was confirmed by the lack of 4sUd incorporation in actD-treated embryos (Additional file 1).

TU-RNA abundance at each time point was measured by microarray analysis, and mRNA half-life was calculated by fitting measurements to a single exponential decay equation. Data for the majority of mRNAs fit this model; 79% of mRNAs from the uridine-only data (UO), and 87% of mRNAs from the uridine plus actD data (U + actD) fit with R^2^ ≥ 0.75. The UO and U + actD data were first used to test for potential biases in decay measurements. We did not detect any correlation between uridine number and mRNA half-life (Additional file 2), demonstrating that uridine frequency does not affect decay measurements. We also found no correlation between transcript abundance and mRNA half-life, demonstrating that there is no bias in the decay measurements for low-abundance versus high-abundance transcripts (Additional file 2).

**Figure 1:**
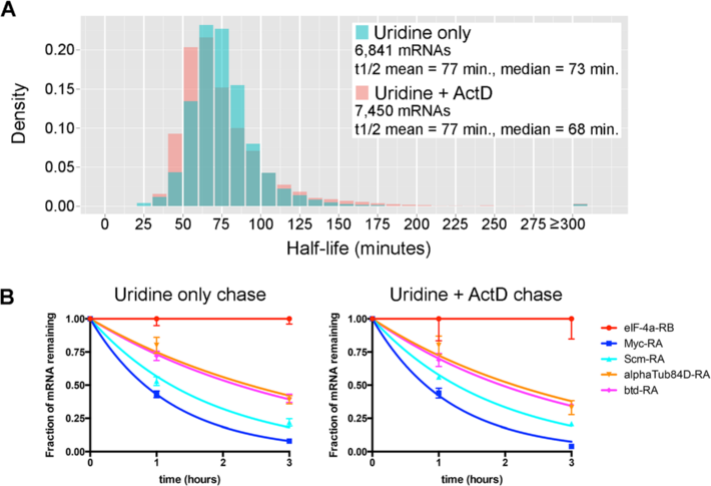
TU-decay measures genome-wide mRNA half-lives without transcription arrest. **(A)** Distribution of whole embryo mRNA half-lives based on uridine chase conditions (Uridine only) and uridine chase combined with actinomycin D to arrest transcription (Uridine + ActD). t_1/2_ = half-life. **(B)** Individual transcript decay curves based on the uridine-only and uridine + actD measurements. Chase times are plotted on the *x*-axis (0-hour = end of 1-hour pulse). Mean chase/pulse values are plotted at the 1-hour and 3-hour time points, and curves were fit using a single-order exponential decay equation. *Error bars* are standard deviation.

The distribution of mRNA half-lives was similar for pulse-chase experiments performed with or without actD (Figure 1A), and decay kinetics for the majority of mRNAs were highly reproducible; only 11.6% of mRNAs had standard deviations in half-life ≥30% of the mean for that transcript. Many transcripts gave nearly identical results with or without actD in the chase media (Figure 1B), including orthologs of known mammalian high-stability mRNAs (for example, *eIF4a* [15]) and low-stability mRNAs (for example, *Myc* [16]). An additional indication of the reproducibility of these measurements is provided by gene ontology (GO) analysis of the UO and U + actD datasets. The top 1,000 most stable transcripts and the bottom 1,000 least stable transcripts from both datasets yielded very similar GO results (Additional file 3). Given the reproducibility and similarity of decay measurements obtained with or without actD, we conclude that TU-decay effectively measures mRNA decay without the need to use pharmacological inhibitors of RNA polymerase II or other methods of arresting transcription.

Gene ontology categories identified by combining the UO and U + actD datasets are summarized in Table 1, and a complete list of half-lives is provided in Additional file 4. The results are similar to category enrichments seen for stable *versus* unstable mRNAs in studies of *Drosophila* maternal mRNA decay [10], yeast mRNA decay [17], and mammalian mRNA decay [18]. High-stability transcripts had a significant enrichment of mRNAs encoding ribosomal proteins, cytoskeletal proteins, chromatin-associated proteins, electron transport proteins, and other mRNAs involved in constitutive or ‘housekeeping’ functions. Low-stability transcripts had a significant enrichment of mRNAs encoding transcription factors, kinases, and other mRNAs associated with cell-fate decisions and organ development, including regulators of neuron projection morphogenesis. This agreement with gene ontology results obtained using alternative model systems further supports the validity of our pulse-chase approach. We conclude that TU-decay is an effective technique for measuring mRNA decay in *Drosophila* embryos.

**Table 1:** **Whole embryo mRNA decay analysis: functional annotation of high-stability and low-stability mRNAs**

**Whole embryo measurements: high-stability mRNAs**					
**GO term**	**Definition**	***P*** **value**	**FDR**	**Count**	**Fold enrichment**
0022626	Cytosolic ribosome	2 × 10^−57^	5 × 10^−56^	67	10.3
	*RpL4*, *RpL23*, *RpS16*, *RpS24*				
0000022	Microtubule cytoskeleton	8 × 10^−17^	3 × 10^−14^	57	3.5
	*Tub84b*, *Tub56D*, *ssp4*, *robl*				
0022900	Electron transport chain	1 × 10^−14^	2 × 10^−12^	29	5.9
	*mt:Cyt-b, mt:CoI*, *mt:CoII*, *mt:ND1*				
0031497	Chromatin assembly	2 × 10^−9^	2 × 10^−7^	15	7.8
	*His1*, *His2A*, *His2Av*, *His2B*, *His3*				
0045941	Positive regulation of transcription	4 × 10^−3^	.14	15	2.4
	*DSIF*, *HP1*, *Trl*, *ash2*, *lid*, *arm*, *btd*				
0048024	Regulation of mRNA splicing	7 × 10^−3^	.19	10	2.9
	*Hrb27c*, *Hrb87f*, *B52*, *SC35*, *snf*				
**Whole embryo measurements: low-stability mRNAs**					
**GO term**	**Definition**	***P*** **value**	**FDR**	**Count**	**Fold enrichment**
0004672	Protein kinase activity	2 × 10^−9^	1 × 10^−6^	41	2.6
	*S6kII*, *BubR1*, *mkk4*, *dTOR*, *wts*, *htl*				
0048812	Neuron projection morphogenesis	2 × 10^−8^	2 × 10^−6^	40	2.7
	*Con*, *trio*, *Ephrin*, *Eph*, *otk*, *drl*, *rl*				
0006351	Transcription	1 × 10^−5^	4 × 10^−4^	47	1.8
	*svp*, *dpn*, *Myc*, *grn*, *kn*, *Trx*, *eve*				
0019226	Transmission of nerve impulse	3 × 10^−5^	9 × 10^−4^	26	2.6
	*CASK*, *cha*, *synaptogyrin*, *cpx*, *shi*				
0014706	Striated muscle tissue development	3 × 10^−5^	1 × 10^−3^	11	5.3
	*Hem*, *Mef2*, *Mlp84B*, *flw*, *mbc*				
0051674	Localization of cell	5 × 10^−4^	.01	23	2.3
	*cbl*, *APC*, *Dl*, *Mhc*, *baz*, *dome*, *cv-c*				

Gene ontology categories enriched among the 500 most stable and 500 least stable transcripts were identified using DAVID. Representative genes are listed for each category. FDR is the Benjamini-Hochberg false discovery rate.

### TU-tagging purifies neural-transcribed mRNAs from whole embryos

**Figure 2:**
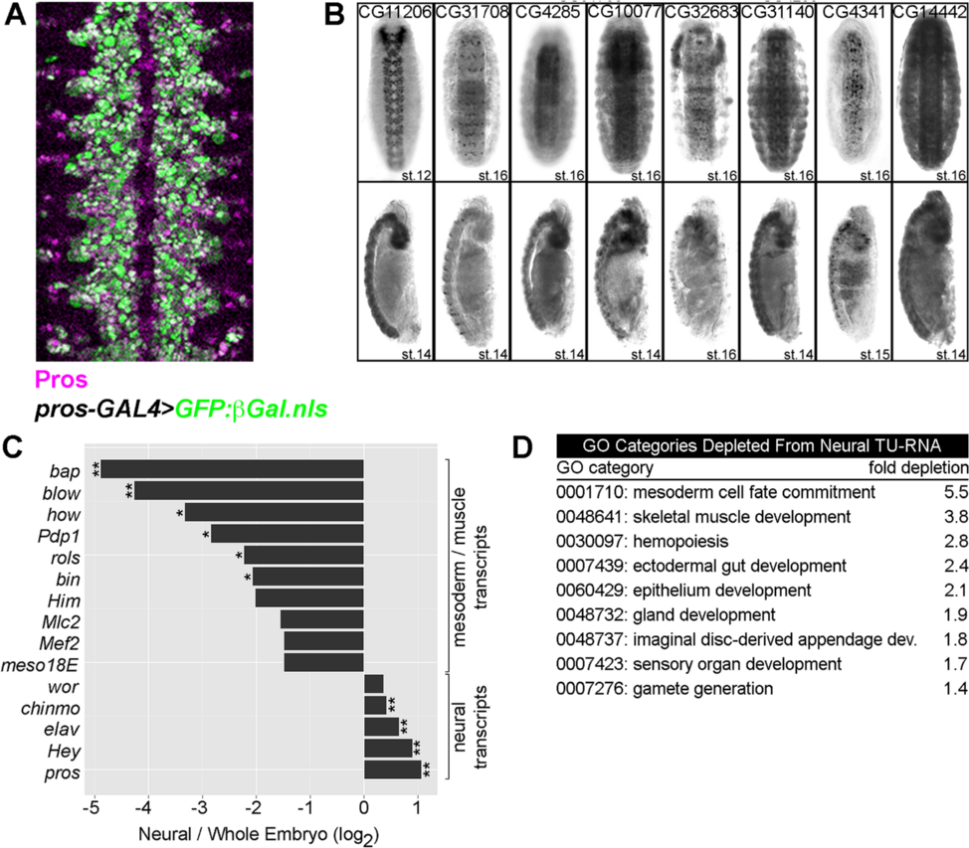
*Pros > UPRT* TU-tagging purifies neural mRNAs from embryos. **(A)** *Prospero-GAL4* driving *UAS-GFP::lacZ.nls* (encoding a nuclear-localized GFP-β-galactosidase fusion) in the embryonic central nervous system shows the expression pattern of *prospero-GAL4*. Anti-Prospero = *magenta*, anti-β-galactosidase = *green*. **(B)** RNA *in situ* hybridization for eight genes identified as nervous system-enriched by TU-tagging. Gene annotation symbols are listed in the top panel. Top panel = ventral view. Bottom panel = lateral view. Developmental stage is listed in the *lower right corner* of each embryo image. **(C)** Relative abundance of mesoderm or muscle-specific mRNAs and neural-specific mRNAs in 1-hour pulse-labeled TU-RNA samples. ** = FDR ≤0.20, * = FDR ≤0.40 based on SAM analysis. **(D)** Gene ontology categories depleted from neural TU-RNA samples (mRNAs below detection in the 1-hour pulse *pros > UPRT* TU-RNA microarrays but present in 1-hour pulse whole embryo TU-RNA microarrays). All categories were significantly depleted at *P* values ≤ 1.0 × 10^−4^ based on DAVID analysis.

We previously used TU-tagging to identify *taranis* as a nervous system-expressed gene in *Drosophila* embryos [19]. These experiments used *prospero-GAL4* to drive *UAS-T.g.UPRT* expression (*pros > UPRT*). Embryos at 0 to 16 h of development were exposed to 4-thiouracil (4TU) for 2 h and TU-tagged RNA was isolated from whole embryo lysates. Purified TU-RNA was compared to non-tagged RNA from the same embryos in microarray experiments. The *prospero-GAL4* construct expresses GAL4 in neuroblasts, ganglion mother cells, and glia of the central nervous system (CNS) (Figure 2A) in addition to peripheral sensory neurons. *Prospero-Gal4* driving *UAS-T.g.UPRT* also results in mRNA tagging in post-mitotic CNS neurons since the UPRT (and likely some converted 4-thio-UMP) is transferred from progenitors to neurons [20].

Analysis of the complete *pros > UPRT* data (not previously described) identified 148 genes enriched 1.5-fold or greater compared to untagged mRNA, including *prospero* itself (Additional file 5). Published literature and the Berkeley *Drosophila* Genome Project (BDGP) *in situ* database provided embryonic expression patterns for 108 of the 148 enriched genes. Of these, 94 (87%) are expressed in the nervous system. To further test the identification of neural mRNAs, we performed *in situ* hybridizations for ten enriched mRNAs whose expression patterns had not previously been characterized. We found that all ten were expressed in the nervous system, with eight specifically expressed in the CNS (Figure 2B). In total, we verified that 104 out of 118 (88%) genes identified by *pros > UPRT* TU-tagging were expressed in the nervous system.

We used the same *pros > UPRT* genotype to perform nervous system-specific TU-decay analysis. Stage 12 to 15 embryos were exposed to the pulse-chase time course described for whole embryos. To confirm enrichment of neural mRNAs in these experiments, we compared the pulse-labeled TU-RNA from *pros > UPRT* embryos to the pulse-labeled TU-RNA from whole embryos. Neural-specific TU-RNA had a modest enrichment of known nervous system-expressed mRNAs, as expected given the presence of these transcripts in both stage-matched samples (Figure 2C). Neural-specific TU-RNA was significantly depleted of many mRNAs primarily expressed in the mesoderm and muscle (Figure 2C). We also performed gene ontology analysis of transcripts that were absent from neural-specific TU-RNA but present in whole embryo TU-RNA. This revealed a significant depletion of transcripts associated with the development or function of tissues outside the nervous system (Figure 2D). Based on the above indicators of nervous system specificity, we proceeded to use the *pros > UPRT* pulse-chase data to calculate neural mRNA half-lives*.*

### Functionally related mRNAs have neural-specific decay kinetics

**Figure 3:**
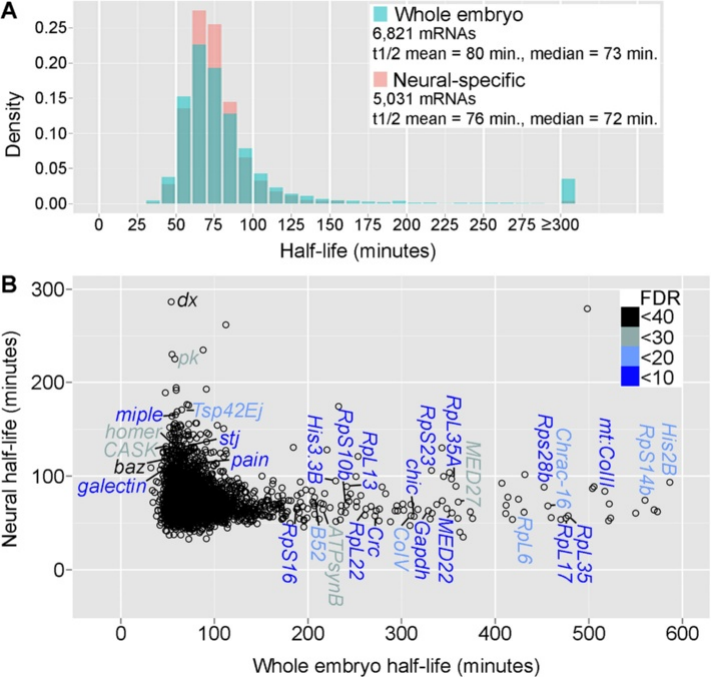
Comparison of neural-specific and whole embryo decay measurements suggests many transcripts have neural-specific half-lives. **(A)** Distribution of mRNA half-lives in the whole embryo and neural-specific datasets. Only mRNAs with reproducible half-life measurements across replicates were included in the distribution and mean/median calculations. **(B)** Comparison of individual mRNA half-lives obtained by neural-specific *versus* whole embryo measurements. *Spots* represent the mean half-life of individual transcripts. Additional mRNAs with whole embryo half-lives estimated at ≥600 min are listed in Additional file 7. Select mRNAs with half-life differences of ≥1.5-fold are labeled, with statistical significance indicated by the FDR color code.

Neural mRNA half-lives were calculated as described for whole embryos. Seventy-three percent of mRNAs fit the exponential decay equation with an R^2^ ≥ 0.75. The reproducibility of neural-specific measurements was similar to that observed for whole embryos; between biological replicates, only 13.1% of mRNAs had standard deviations in half-life ≥30% of the mean for that transcript. The distribution of mRNA half-lives based on neural-specific and whole embryo measurements was very similar (Figure 3A). This suggests that the general mRNA-decay machinery and kinetics of turnover are not substantially different in the nervous system compared to other tissues. Genome-wide neural-specific mRNA half-lives are provided in Additional file 6.

Gene ontology analysis of low-stability versus high-stability neural mRNAs identified several differences compared to the whole embryo dataset (Table 2). While both datasets had the category ‘transcription’ enriched among low-stability mRNAs, the neural-specific measurements had a unique enrichment of mRNAs involved in neuron differentiation, mitosis, histone methylation, endocytosis, and ribosome biogenesis. Differences between the two datasets are even more striking for high-stability mRNAs. The most significantly enriched category for neural high-stability mRNAs is ‘neuron projection morphogenesis’ which includes regulators of axonogenesis and axon pathfinding. In contrast, mRNAs in this category were among the least stable mRNAs in whole embryos, suggesting differential regulation of some transcripts within this GO category. Examples of ‘neuron projection morphogenesis’ mRNAs with inverse stabilities based on neural-specific *versus* whole embryo measurements include *APC-like*, *Abelson interacting protein*, and *off-track.* Other high-stability mRNAs unique to the neural-specific dataset include those encoding small GTPases that regulate vesicle trafficking and transcripts encoding regulators of programmed cell death.

**Table 2:** **Neural-specific mRNA decay analysis: functional annotation of high-stability and low-stability mRNAs**

**Neural-specific measurements: low-stability mRNAs**					
**GO term**	**Definition**	***P*** **value**	**FDR**	**Count**	**Fold enrichment**
0006351	Transcription	4 × 10^−10^	2 × 10^−7^	58	2.5
	*svp*, *dpn*, *pros*, *vvl*, *grh*, *gsb*, *onecut*				
0030182	Neuron differentiation	2 × 10^−7^	4 × 10^−5^	48	2.3
	*chinmo*, *Dl*, *tup*, *Ephrin*, *Eph*, *fax*, *shot*				
0007067	Mitosis	6 × 10^−5^	3 × 10^−3^	21	2.9
	*cycB3*, *cdc27*, *Sse*, *cnn*, *glu*, *mts*				
0018024	Histone methyltransferase activity	2 × 10^−4^	.02	7	6.9
	*G9a*, *Set1*, *Set2*, *Su(z)12*, *ash1*, *egg*				
0006897	Endocytosis	4 × 10^−3^	.08	25	1.9
	*Arpc4*, *shi*, *CalpA*, *pat1*, *faf*, *scb*, *Rac1*				
0005840	Ribosome	2 × 10^−3^	.06	21	2.0
	*RpL17*, *RpL35*, *mRpL11*, *mRpS35*				
**Neural-specific measurements: high-stability mRNAs**					
**GO term**	**Definition**	***P*** **value**	**FDR**	**Count**	**Fold enrichment**
0048812	Neuron projection morphogenesis	5 × 10^−6^	6 × 10^−4^	33	2.4
	*trc*, *fusl*, *Fas2*, *Appl*, *Fmr1*, *otk*, *ben*				
0003924	GTPase activity	2 × 10^−4^	.02	17	2.9
	*Rab23*, *Rab30*, *Rab11*, *Rho1*, *Ras64B*				
0012501	Programmed cell death	2 × 10^−3^	.06	17	2.4
	*grim*, *pten*, *scyl*, *egr*, *mod(mdg4)*				
0004672	Protein kinase activity	9 × 10^−3^	.24	23	1.8
	*CamKI*, *Cdk4*, *Ror*, *ald*, *aur*, *nmo*, *trbl*				
0019226	Transmission of nerve impulse	.02	.25	17	1.8
	*CASK*, *CanB*, *Dap160*, *cpx*, *Sap47*				
0051674	Localization of cell	.01	.16	18	1.9
	*cbl*, *APC*, *cortactin*, *par-6*, *baz*, *spri*				

Gene ontology categories enriched among the 500 most stable and 500 least stable transcripts were identified using DAVID. Representative genes are listed for each category. FDR is the Benjamini-Hochberg false discovery rate.

Genome-wide comparison of mRNA half-lives between the neural-specific and whole embryo datasets revealed that 24% of mRNAs had statistically-significant half-life differences of ≥1.5-fold (484 mRNAs had decreased stability based on neural-specific measurements, and 409 mRNAs had increased stability based on neural-specific measurements, out of 3,776 transcripts) (Figure 3B and Additional file 7). Importantly, these differences do not randomly occur across the transcriptome; mRNAs with different half-lives based on neural-specific *versus* whole embryo measurements fall into specific gene ontology categories (Table 3). mRNAs with shorter half-lives based on neural-specific measurements include those encoding ribosomal proteins (26 ribosomal protein mRNAs), translation regulators (*EF2*, *eIF-5A*), histones (7 histone mRNAs), and metabolic enzymes (*Gapdh1*, multiple ATP synthetases). mRNAs with longer half-lives based on neural-specific measurements include those involved in neurotransmission (*CASK*, *stj*, *homer*, *Dap160*, *Rop*), axonogenesis (*pk*, *Appl*, *miple*, *galectin*), axonal transport (*Klp64D*, *TBCB*), and establishment or maintenance of neuroblast polarity (*baz*, *par-6*, *Lgl*, *pins*, *G*α*o*). Each of these genes is transcribed in the nervous system plus additional tissues (based on FlyBase annotations and/or BDGP *in situ* data), suggesting that half-life differences between whole embryo and neural-specific measurements are due to neural-specific mRNA decay kinetics.

**Table 3:** **Functional annotation of mRNAs with altered stability based on neural-specific versus whole embryo measurements**

**mRNAs stabilized in the nervous system (neural/whole embryo half-life >1.5)**					
**GO term**	**Definition**	***P*** **value**	**FDR**	**Count**	**Fold enrichment**
007409	Axonogenesis	2 × 10^−3^	.06	13	2.8
	*APC*, *CadN*, *trio*, *caps*, *fray*, *otk*, *daw*				
0003924	GTPase activity	2 × 10^−3^	.31	10	3.5
	*Rab21*, *Rab30*, *Ras64B*, *RabX1*				
0009312	Oligosaccharide biosynthesis	9 × 10^−3^	.15	4	9.2
	*mgat2*, *GalNAc-T1*, *pgant2*, *pgant5*				
0045196	Establishment of neuroblast polarity	.01	.16	3	18.7
	*par-6*, *baz*, *Lgl*				
0007269	Neurotransmitter secretion	.04	.56	6	2.6
	*CanB*, *Lgl*, *CASK*, *Dap160*, *cpx*, *rop*				
**mRNAs destabilized in the nervous system (neural/whole embryo half-life <0.6)**					
**GO term**	**Definition**	***P*** **value**	**FDR**	**Count**	**Fold enrichment**
0003735	Structural constituent of ribosome	5 × 10^−17^	5 × 10^−15^	26	9.2
	*RpL11*, *RpL30*, *RpL6*, *RpS16*, *RpS23*				
0000278	Mitotic cell cycle	4 × 10^−8^	4 × 10^−5^	30	3.2
	*dap*, *cycB3*, *ball*, *glu*, *eff*, *ncd*, *mts*				
0031497	Chromatin assembly	3 × 10^−4^	.02	7	7.5
	*H2B*, *H3*, *H3.3A/B*, *HP2*, *Df31*, *Set*				
0005839	Proteasome core complex	8 × 10^−3^	.07	5	6.0
	*Pros26*, *Pros*α*7*, *Pros*β*2*, *Pros*β*7*				
0006119	Oxidative phosphorylation	6 × 10^−4^	.03	12	3.5
	*ATPsynB*, *ATPsynD*, *mt:CoIII*				

Gene ontology categories were identified using DAVID. Representative genes are listed for each category. FDR is the Benjamini-Hochberg false discovery rate.

### Neural mRNA stability correlates with predicted transcript localization and function

Gene ontology analysis of neural mRNA-decay data revealed that mRNAs encoding transcription factors tend to be rapidly decayed, and mRNAs encoding proteins involved in neuron projection morphogenesis and neurotransmission tend to be stable. The rapid decay of transcription-factor mRNAs could be related to transient protein expression during cell-fate specification. To test this hypothesis, we identified 39 mRNAs encoding transcription factors that are involved in CNS neurogenesis (based on Interactive Fly annotations [21]). The stability of mRNAs associated with neuron projection morphogenesis and neurotransmission could be related to their localization in axon growth cones or dendrites. To test this hypothesis, we identified 35 *Drosophila* orthologs of mRNAs that are localized to axon growth cones [22] or dendrites [23] in mammals.

**Figure 4:**
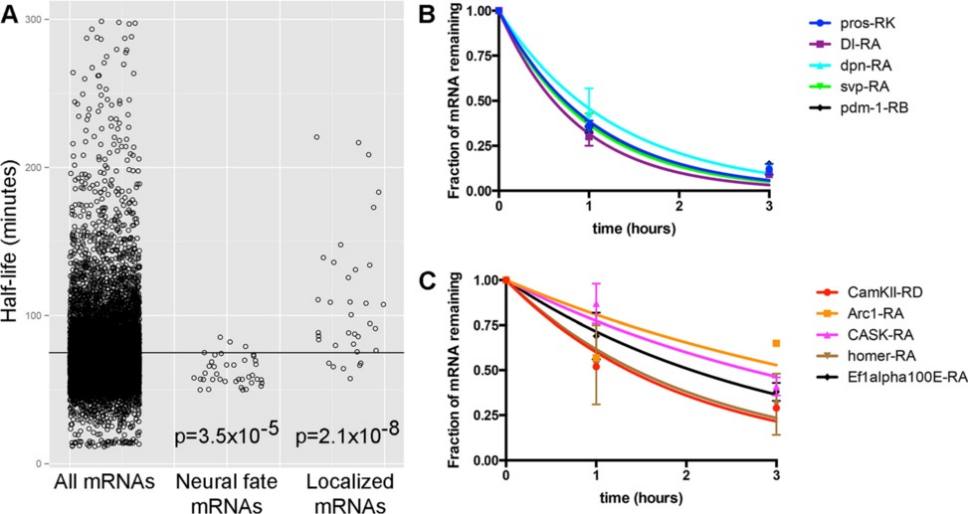
mRNA stability correlates with function and predicted localization. **(A)** mRNA half-life distributions of all neural mRNAs, the neural fate transcription factor subset (‘neural fate’), and the predicted axon growth cone or dendrite-localized mRNA subset (‘localized’). The *horizontal line* marks the mean neural-specific half-life (76 min). *P* values are from Mann-Whitney *U* tests comparing the distribution of half-lives for each subset to the ‘all mRNAs’ set of transcripts. **(B)** Representative decay curves for neural fate mRNAs. **(C)** Representative decay curves for predicted localized mRNAs. For the graphs in B and C, chase times are plotted on the *x*-axis (0-hour = end of 1-hour pulse). Mean and standard deviation chase/pulse values at the 1-hour chase and 3-hour chase time points are plotted based on multiple measurements across biological replicate experiments.

We compared the half-lives of the neurogenesis transcription factor mRNAs (which we refer to as ‘neural fate’ mRNAs) and the half-lives of the predicted axon or dendrite-localized mRNAs (which we refer to as ‘localized’ mRNAs) to the half-lives of all neural mRNAs. This revealed a significant trend of below average stability for neural fate mRNAs and above average stability for localized mRNAs (Figure 4A, Additional file 8). Rapidly decayed neural fate mRNAs include those encoding proteins whose expression is tightly coupled to the timing of neuroblast cell divisions (*cas*, *svp*, *pdm-1*) [24]; proteins that are differentially expressed within neuroblast lineages (*Hey*, *Dl*) [25]; and proteins that are differentially expressed among neuroblasts, GMCs, and neurons (*dpn*, *pros*) [26] (Figure 4B). Thus, rapid mRNA decay correlates with dynamic temporal or spatial patterns of protein expression. Additional transcription factor mRNAs that are rapidly decayed but were not included in the list of neurogenesis transcription factors include *fkh*, *pnt*, *glu*, *BarH1*, and others that may have functions in neural development that require precise regulation of mRNA abundance.

Stable mRNAs predicted to localize to axon growth cones or dendrites include a known *Drosophila* dendrite-localized mRNA (*CamKII* [27]) and two orthologs of mammalian mRNAs that are locally translated in dendrites (*Arc1* [28] and *Ef1*α*100E* [29]) (Figure 4C). Stable predicted localized mRNAs also include transcripts encoding proteins that localize to pre- or post-synaptic sites in *Drosophila* neurons (*homer* [30], *CASK* [31], and *synaptogyrin* [32]), although the site of translation of these mRNAs is unknown. We conclude that the elevated stability of these mRNAs may be related to evolutionarily conserved patterns of subcellular mRNA localization.

### Pumilio regulates neural mRNA decay

To identify sequence elements that might target neural fate mRNAs for rapid decay, we searched for known *cis*-regulatory elements in the 3’ UTRs of this group of transcripts. As a first step, we tested for any correlation between 3’ UTR length and mRNA stability, since shorter 3’ UTRs could contain fewer *cis*-elements. We found no correlation between 3’ UTR length and stability and no bias toward short or long 3’ UTRs in the stable localized *versus* unstable neural fate mRNAs (or in the entire neural mRNA decay dataset) (Additional file 9).

**Figure 5:**
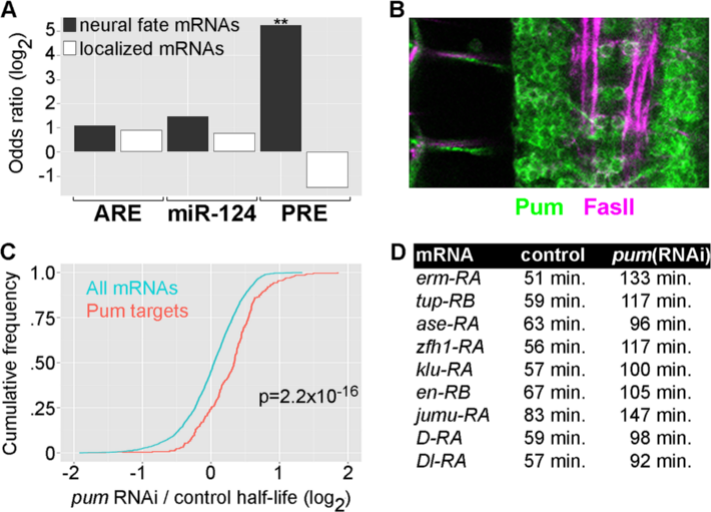
Pumilio regulates neural mRNA stability. **(A)** Enrichment or depletion of candidate *cis*-regulatory elements as follows: AREs, miR-124 target sites, and PREs. Odds ratios were calculated as the frequency of each element within the low-stability (t_1/2_ ≤ 75 min) ‘neural fate’ mRNAs or high-stability (t_1/2_ ≥ 80 min) ‘localized’ mRNAs *versus* the frequency of that element in all low-stability or high-stability neural mRNAs. ** = *P* value < 1.0 × 10^−15^ (Fisher’s test). **(B)** Pumilio expression in a stage 15 embryo detected with an anti-Pumilio antibody (*green*). Anti-Fas2 antibody stain (*magenta*) shows a subset of longitudinal axon fiber tracts and axons leaving the ventral nerve cord. **(C)** Cumulative distribution function plots of mRNA half-life changes in *Pumilio* RNAi *versus* control embryos (*Pumilio* RNAi/control ratio). The distribution for all neural mRNAs is plotted in *blue*. The distribution for predicted Pumilio targets is plotted in *red*. The shift to the right for predicted Pumilio targets indicates a trend of stabilization in *Pumilio* RNAi embryos. The *P* value is from a Kolmogorov-Smirnoff test comparing the half-life changes (*Pumilio* RNAi/control) for predicted Pumilio targets and non-targets. **(D)** Examples of predicted neural fate Pumilio targets with increased mRNA stability in *Pumilio* RNAi embryos. These transcripts had reproducible changes in half-life with FDRs ≤0.30 (based on SAM analysis).

Candidate *cis*-elements were selected based on known roles in maternal mRNA decay, known or predicted expression of the corresponding *trans*-acting factor in the nervous system, and the availability of databases that identify transcripts containing the *cis*-element. Based on these criteria, we tested for enrichment or depletion of AU-rich elements (AREs) [33], miR-124 seed sequences (miR-124 is a nervous system-specific microRNA [34]), and Pumilio recognition elements (PREs) [35]. Of these candidates, only PREs were significantly enriched among the low-stability neural fate mRNAs (20 out of 36 mRNAs with half-life ≤75 min contain a PRE) and depleted from high-stability localized mRNAs, although not at a statistically-significant level (1 out of 29 mRNAs with half-life ≥80 min contain a PRE) (Figure 5A, Additional file 8). AREs and miR124 seed sequences had no significant enrichment or depletion in neural fate or localized mRNAs (Figure 5A) or in the entire set of low or high-stability neural mRNAs.

For the PRE analysis, we used all mRNAs that contain the 8-nucleotide motif UGUA(A/U/C)AUA at least once in their 3’ UTR, as identified by Gerber et al. [35]. Of the 1,431 mRNAs that contain this motif, 439 were present in our neural-specific dataset. PREs do not appear to be a general determinant of neural mRNA stability; there was no significant enrichment of PRE-containing transcripts among all low-stability or high-stability neural mRNAs (8.5% of mRNAs with half-life ≤75 min contain a PRE, and 8.8% of mRNAs with half-life ≥80 min contain a PRE). In addition to mRNAs containing the PRE motif, Gerber *et al*. identified 156 potential Pumilio targets using a tandem-affinity purification (TAP)-tagged Pumilio approach [35]. The list of Pumilio targets identified by TAP does not include any transcripts from our neural fate or localized mRNA classes. Similar to the PRE data, the Pumilio TAP data were not a predictor of mRNA stability among all neural mRNAs (0.9% of mRNAs with half-life ≤75 min were predicted targets by TAP, and 1.5% of mRNAs with half-life ≥80 min were predicted targets by TAP). We conclude that the presence of a PRE motif (and not identification as a Pumilio target by TAP) correlates with low mRNA stability only in the neural fate mRNA class.

The significant enrichment of PREs among low-stability neural fate mRNAs suggested a novel role for Pumilio in neural development. Pumilio has been implicated in the regulation of pre- and post-synaptic morphology and function [36] and memory formation [37] in *Drosophila*. We investigated the expression of Pumilio in the embryonic nervous system and found widespread localization of Pumilio in the cytoplasm of neural progenitors, neurons, and certain axon projections throughout the ventral nerve cord (Figure 5B), similar to Pumilio expression patterns in the larval central nervous system [36].

To test if Pumilio affects neural mRNA decay, we used neural-specific RNA interference [38] to decrease Pumilio expression. Western blot analysis showed that Pumilio RNAi decreased Pumilio protein to approximately 50% of wild-type levels (Additional file 10). Knockdown of Pumilio using *pros-Gal4* X *UAS-pumilio{RNAi}* did not cause detectable neural development defects, based on analysis of Fas2^+^ axon projection patterns and production of Eve^+^ neurons. We conclude that partial knockdown of Pumilio has no effect or subtle effects on neural development. However, we predicted that partial knockdown of Pumilio should be sufficient to induce changes in mRNA stability if Pumilio regulates mRNA decay.

We performed TU-decay experiments in Pumilio knockdown embryos, with *pros-GAL4* driving *UAS-Pumilio{RNAi}* and *UAS-T.g.UPRT*. Neural mRNA half-lives in the Pumilio knockdown embryos were compared to control *pros > UPRT* half-lives. Reproducible half-life measurements were obtained for 3,008 transcripts, and predicted Pumilio targets (PRE-containing transcripts) showed a significant trend of stabilization compared to non-targets (Figure 5C, Additional file 11). Data were obtained for 265 predicted Pumilio targets, and 132 (50%) had a statistically-significant increase in mRNA half-life of ≥1.3-fold. As expected, many of the stabilized Pumilio targets encode transcription factors and other cell-fate determinants (Figure 5D). Stabilized Pumilio targets also include regulators of axon pathfinding (*Eph receptor tyrosine kinase*, *off-track*, *Sema-1a*) that are not predicted to localize in axon growth cones. These experiments did not yield reproducible data for the one stable Pumilio target predicted to localize to axon growth cones or dendrites, *Tetraspanin 42Ee*. However, we did obtain measurements for another predicted localized Pumilio target, *discs large 1* (*dlg1*). *dlg1* mRNA was previously identified as a target of translation repression by Pumilio [39], and its ortholog, PSD-95, is localized to dendrites in mammalian neurons [40]. *dlg1* mRNA is relatively unstable in control embryos (half-life =65 min) and is significantly stabilized in *Pumilio{RNAi}* embryos (half-life =127 min).

**Figure 6:**
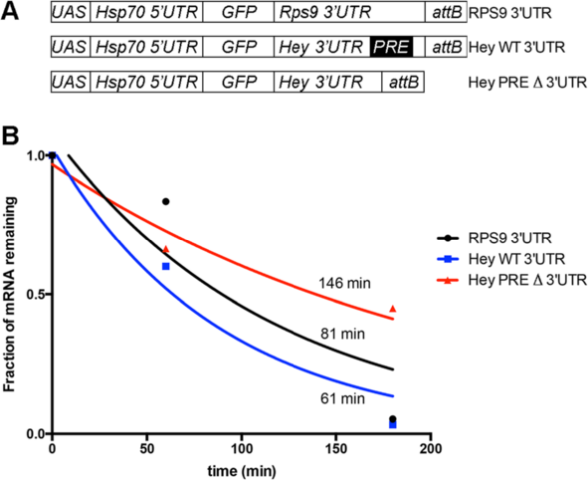
PREs increase the rate of transcript decay in the nervous system. **(A)** Reporter construct design. Hey WT 3’ UTR contains the single endogenous PRE. Hey PRE Δ 3’ UTR has only the PRE sequence removed. **(B)** Decay curves based on reverse-transcription, quantitative-PCR measurements of reporter transcript decay (using GFP primers). Chase times are plotted on the *x*-axis (0-hour = end of 1-hour pulse). Mean chase/pulse values at the 1-hour chase and 3-hour chase time points are plotted based on multiple measurements. Half-lives in minutes are listed next to the decay curve for each transcript.

To test if a 3’ UTR PRE is necessary for destabilization of a predicted Pumilio target in the nervous system, we constructed reporter transcripts composed of the *hsp70* 5’ UTR, the green fluorescent protein (GFP) open reading frame, and one of three 3’ UTR sequences containing an endogenous transcription termination signal; the *RPS9* 3’ UTR (without any PRE), the *Hey* 3’ UTR (containing a single naturally occurring PRE), and a *Hey* 3’ UTR with the PRE deleted (Figure 6A). These reporter constructs were placed under *UAS* control and integrated into the same chromosome locus in transgenic fly lines. *Pros-Gal4* was used to activate transcription of the reporter and *UAS-UPRT*, thus allowing neural-specific measurement of reporter stability. The reporter containing the RPS9 3’ UTR had slightly above average stability, similar to the endogenous RPS9 transcript (Figure 6B). In contrast, the reporter containing the Hey 3’ UTR was rapidly decayed. Deletion of the PRE from the Hey 3’ UTR caused a dramatic increase in stability compared to the unmodified Hey 3’ UTR reporter. These results agree with the increased stability of the endogenous Hey transcript in *Pumilio{RNAi}* embryos and the lack of any significant change in endogenous RPS9 transcript stability in *Pumilio{RNAi}* embryos (Additional file 11). We conclude that the single PRE within the Hey 3’ UTR targets this transcript for degradation in the nervous system.

## Discussion

mRNA abundance during neural development is determined by the rate of gene transcription and the rate of mRNA decay. We addressed the role of mRNA decay in neural development using the TU-decay technique. Our results provide *in vivo* measurements of zygotic mRNA decay in *Drosophila* embryos and allow investigation of neural-specific mRNA decay properties. It is difficult to determine how closely half-lives calculated by TU-decay reflect endogenous decay rates, since any technique used to quantify mRNA turnover may introduce biases. However, the global trends of mRNA decay identified in this work are similar to those described for other systems (for example, gene ontology categories for low-stability *versus* high-stability mRNAs), suggesting that TU-decay provides accurate measurements of relative mRNA stability.

Our comparison of whole embryo and neural-specific mRNA decay data revealed unique neural mRNA decay properties for 893 transcripts. While we have not checked the expression patterns of all 893 transcripts, many are expressed in the nervous system and additional tissues. We conclude that the different half-life calculations for these transcripts are due to heterogeneous transcript decay measurements in whole embryo experiments *versus* more homogeneous transcript decay measurements in the neural-specific TU-decay experiments. Neural-specific decay properties likely enable a single mRNA to have distinct expression dynamics in the nervous system compared to other tissues.

Many transcripts with decreased stability in the nervous system encode proteins involved in cell growth and proliferation, such as ribosomal proteins and histones. This may relate to the transition from proliferation to differentiation that occurs in the nervous system at the embryonic stages we analyzed. Early-born neuroblast progeny differentiate into neurons and glia at stage 12, and progenitor proliferation significantly decreases by stage 15 [41]. A similar decrease in proliferation occurs in yeast shifted to nutrient-limited media, and this transition causes a dramatic destabilization of ribosomal protein mRNAs [13]. Lower histone mRNA stability has been described for human fibroblasts compared to their corresponding induced pluripotent stem cells (iPSCs) [42]. Histone mRNAs are more stable in iPSCs, presumably to support chromosome replication and the production of differentiated progeny. Similar destabilization of histone mRNAs in the differentiating embryonic nervous system may be related to the decreased mitotic potential of progenitors and the post-mitotic state of neurons.

Many transcripts with increased stability in the nervous system encode proteins with known neural-specific functions, including neurotransmission, axonogenesis, and maintenance of neuroblast polarity. Many of these genes have widespread expression patterns but elevated levels of mRNA or protein in the nervous system. Examples include *Cadherin-N* (*CadN*), *Apc-like* (*Apc*), and *bazooka* (*baz*). CadN is expressed in embryonic muscles and neurons, but the protein is detected at much higher levels in neurons [43]. Apc is detected ubiquitously at low levels in embryos, with high levels of protein concentrated in the central nervous system [44]. *Bazooka* mRNA is detected in epithelial cells and neuroblasts, where Bazooka regulates spindle orientation and cell polarity [45]. Multiple mRNAs encoding regulators of neuroblast polarity had increased stability based on neural-specific measurements as follows: *baz*, *pins*, *par-6*, *Lgl*, and *G*α*o*. The stability of these transcripts may support appropriate levels of protein production in neuroblasts without the need for high rates of transcription. Baz, Par-6, and Lgl also have functions in post-mitotic neurons [46,47] and the increased stability of these mRNAs could be due to stabilization in neurons.

Genome-wide studies of mRNA decay in other systems support a model in which mRNA half-life is linked to the function of the encoded protein [10,48]. We found that this model is applicable to mRNAs encoding transcription factors that regulate neural cell-fate decisions. These transcription factors tend to act transiently in neural progenitors or their progeny and have precise spatial or temporal expression patterns. While the expression of these genes likely involves transcriptional induction and repression, a short mRNA half-life will ensure robust expression patterns. Many cell-fate determinant transcription factors appear to be transcribed at low levels and rapidly decayed, since 1-hour pulse measurements yielded weak signals, and we could not detect any signal above background in subsequent chase measurements. Examples include cell-fate determinants that are transiently expressed in neural progenitors, such as *gcm* [49], *ac* [50], and *Kr* [51]. Future work could improve the analysis of weakly transcribed, rapidly decayed mRNAs by including earlier chase time points and using more sensitive detection methods (such as RNA-sequencing).

We also identified a potential relationship between mRNA half-life and mRNA localization in neurons. While the predicted localization of candidate mRNAs remains to be confirmed, several of the corresponding proteins are known to localize to axon terminals or dendrites in *Drosophila*. It will be interesting to test if a long mRNA half-life is predictive of subcellular localization in neurons, particularly for transcripts whose mammalian orthologs have not previously been identified in growth cones or dendrites. For example, *Stretchin-Mlck* and *Fuseless* are among the most stable neural mRNAs, and their proteins have known roles in axon guidance [52] and synaptic vesicle exocytosis [53], respectively. Stretchin-Mlck is predicted to act at axon growth cones, and Fuseless is concentrated in presynaptic sites of neuromuscular junctions, but their mRNA localization and site of translation is unknown.

While the data generated in this study may be useful for *de novo* identification of *cis*-regulatory elements, here we chose to focus on candidate elements that might determine the decay rate of neural cell-fate mRNAs. Pumilio recognition elements were the only significantly enriched element among this class of mRNAs. Traditional models of Pumilio function emphasize its role as a negative regulator of translation, but recent work has shown that *Drosophila* Pumilio and human orthologs promote deadenylation that is likely to subsequently trigger transcript decay [54]. In addition, an mRNA decay function for Pumilio was predicted based on the enrichment of PREs in *Drosophila* maternal mRNAs degraded by zygotic factors [10]. In this study, we identified a novel mRNA decay function for Pumilio during neural development. We found that an 8-nucleotide PRE motif defined by Gerber *et al*. [35] is significantly enriched among low-stability neural fate mRNAs and is necessary for decay of a reporter construct. Interestingly, PRE-containing neural fate mRNAs were not previously identified as Pumilio targets by tandem-affinity purification using a TAP-tagged Pumilio fragment [35]. The absence of these transcripts from the TAP dataset may be due to differences in experimental design. For example, the TAP experiments used a maternal GAL4 to drive expression of TAP-Pumilio ubiquitously in early embryos while our TU-decay data are neural-specific. These results may also indicate that Pumilio-dependent mRNA decay involves co-factors that are neural-specific. The increased stability of neural fate mRNAs in Pumilio knockdown embryos supports our model and suggests that Pumilio influences critical programs of gene expression during neural development. The regulation of Pumilio-dependent mRNA decay in the developing nervous system will be an important area of future investigation.

## Conclusions

TU-decay experiments revealed dynamic regulation of mRNA stability within the developing nervous system. These results suggest that differential mRNA decay contributes to the spatiotemporal gene expression patterns for many important regulators of neural development. These data provide a useful resource for defining components of the neural mRNA decay regulatory network, as demonstrated by our identification of Pumilio as an mRNA stability determinant.

## Methods

### Fly lines

The following fly lines were used: *UAS-T.g.UPRT* (3rd chromosome), *UAS-T.g.UPRT* (2nd chromosome, provided by M. O’Connor), *prospero-Gal4*, and *UAS-GFP::lacZ.nls (*Bloomington Drosophila Stock Center (BDSC))*.* Whole embryo TU-decay experiments were performed using embryos of either *UAS-T.g.UPRT* line without any Gal4 driver. Pumilio knockdown was performed using the TRiP project line HMS01685 (BDSC stock number 38241). *UAS-GFP-3’UTR* reporter constructs were made by modifying *pUAST-3XemGFP* (*Drosophila* Genetics Resource Center) to remove two copies of emGFP and replace the *SV40* terminator with the *Drosophila Hsp70* terminator and an *attB* site. *RPS9*, *Hey* wild type, and *Hey* PRE deletion 3’ UTR DNA were chemically synthesized (Integrated DNA Technologies) and ligated into the modified *pUAST-emGFP-attB* backbone. Constructs were injected into *P{nos-phiC31\int.NLS}X, P{CaryP}attP40* embryos for PhiC31-mediated integration (Rainbow Transgenic Flies, Inc.).

### TU-tagging

Stage 12 to 15 embryos were used for pulse-chase labeling of RNA. Embryos were permeabilized as previously described [12] and pulse-labeled in tagging media [D22 insect media, 5% FBS, and either 1 mM 4-thiouridine (whole embryo tagging) or 1 mM 4-thiouracil (neural-specific tagging)] for 1 h at 30°C. Pulse-labeled embryos were then transferred for 1 or 3 h to chase media containing uridine [D22 insect media, 5% FBS, 10 mM uridine] at 30°C. Embryos were homogenized for 30 sec and frozen in Trizol (Invitrogen) at −80°C. Multiple samples for each time point were pooled for RNA extraction and TU-RNA purification, as previously described [12].

### Microarray analysis

Biological replicates (independent RNA collections, TU-RNA purifications, and microarray hybridizations) were performed for all experiments. Purified TU-RNA plus a spike-in control (One-Color RNA Spike-In Kit, Agilent) was used to generate Cy3 labeled cRNA (Low Input Quick Amp Labeling Kit, Agilent) and hybridized to Agilent 4x44K *Drosophila* gene expression microarrays. Signal intensities within a pulse-chase series were normalized using the spike-in control. For comparisons between pulse-chase series, 1-hour chase/1-hour pulse and 3-hour chase/1-hour pulse ratios were normalized so that the mean of the ratios at each time-point was the same across experiments (the top and bottom 10% ratio values were excluded from normalization factor calculations). For comparisons of signal intensities in 1-hour pulse samples (Figure 2C), spots with values below background were excluded from mean normalization calculations but were included in calculations of average signal intensity per gene.

### Data access

The microarray data were deposited in the NCBI Gene Expression Omnibus (GEO) (http://www.ncbi.nlm.nih.gov/geo/) under accession numbers GSE67435 (whole embryo data) and GSE67512 (neural-specific data).

### Statistical analysis

Statistical analyses of genome-wide mRNA levels and genome-wide half-life values were made using mean-normalized ratio values for each dataset and absolute difference calculations using the significance analysis of microarrays (SAM) program [55]. Two-class, unpaired sample analysis and the T-statistic were used to calculate *q* values (a multiple testing corrected false discovery rate (FDR)). FDR values listed in figure legends are the *q* value (for statistical analyses performed using SAM). All other statistical analyses were made using the R software environment. For comparisons of half-life values between samples (neural-specific *versus* whole embryo, *Pumilio* RNAi *versus* control), only transcripts with reproducible measurements across biological replicates were included. Reproducible measurements were defined as those with standard deviation in half-life ≤60% of the mean half-life.

### mRNA half-life calculations

Normalized signal intensities for time 0, 1, and 3 h were fitted to the linear transform of a single order exponential decay model, ln(Ε(*t*)) = Ε_0_^*kt*^, by least squares regression analysis in R. E_0_ is the transcript expression value for the pulse-labeled sample and *k* is the decay rate constant. Fit of the decay model was assessed by an adjusted R^2^ value. Half-life was determined by the equation, t_1/2_ = ln2/*k*. Many mRNAs whose levels decreased slightly or not at all during the chase had R^2^ ≤ 0.75. These high-stability mRNAs (typically with half-lives estimated at ≥300 min) were excluded from calculations of mean and median half-life for all datasets. mRNAs in this category that had reproducible half-life measurements across biological replicates (standard deviation/mean ≤60%) were included as high-stability mRNAs for gene ontology analyses and comparisons of whole embryo *versus* neural mRNA half-lives.

### Gene ontology analysis

Gene ontology analysis was performed using DAVID functional annotation clustering [56,57], with default settings and high classification stringency. Unnamed genes (those identified by CG number only) were excluded from GO analysis. Representative GO terms for each annotation cluster are listed in the results tables. Functionally redundant annotation clusters were excluded.

### Immunofluorescent imaging and *in situ* RNA hybridization

Embryo fixation and antibody staining were performed using standard methods. The following antibodies were used: rat anti-Pumilio (1:200, provided by Robin Wharton), mouse anti-Fas2 (1:100, Developmental Studies Hybridoma Bank), mouse anti-prospero (1:20, Developmental Studies Hybridoma Bank), rabbit anti-β-gal (1:1000, Abcam). RNA probes were made using cDNA clones obtained from the *Drosophila* Genome Resource Center, Indiana University, USA. Digested plasmids were used as template for *in vitro* transcription of DIG-labeled RNA probes (Roche Applied Science). *In situ* hybridization was performed using standard methods.

### Reverse transcriptase quantitative PCR

Purified TU-RNA quality was assessed by Bioanalyzer RNA Pico Eukaryotic total RNA assay (Agilent) prior to whole transcriptome amplification and cDNA synthesis (MessageBOOSTER Whole-TranscriptomecDNA Synthesis Kit for qPCR, Epicentre). Real-time PCR quantitation was performed on a Rotor-Gene Q (Qiagen) in 20 uL reactions using QuantiTect Primer Assays (Qiagen) or custom designed primers (table), and SYBR green detection (SYBR Select Master Mix, Applied Biosystems). *Ct* values were normalized to an *RpL32* internal reference and relative abundance calculated by the equation, fold-change = 2^Δ(Δ*Ct*)^. Transcript decay was modeled as described above using relative abundance at time 0, 1 and 3 h.

## Additional files

Additional file 1:
**Transcription arrest in actinomycin D treated embryos.** TU-RNA blot for control and ActD-treated embryos. Additional file 2:
**Uracil number and transcript abundance do not bias mRNA decay measurements.** Scatter plots of uracil number versus mRNA half-life (A) and transcript abundance in the 1-hour pulse sample *versus* mRNA half-life (B). Additional file 3:
**Gene ontology categories of low-stability and high-stability mRNAs from UO and U + actD experiments.** Table listing the gene ontology category enrichment for the 1,000 least stable and 1,000 most stable mRNAs in each dataset. Additional file 4:
**Whole embryo mRNA 1-hour pulse expression levels and transcript half-lives.** Table listing normalized 1-hour pulse (t0) microarray signal intensity and normalized transcript half-life. Values are the mean per transcript calculated by combining the UO and U + actD experiments. Standard deviation (SD) is listed for the 1-hour pulse and half-life values. Additional file 5:
**Neural-enriched mRNAs identified by Pros > UPRT TU-tagging.** Table listing log_2_ ratio of normalized microarray signals for TU-RNA purified from Pros > UPRT embryos divided by untagged RNA signals from the same embryos (pros UPRT log_2_ 4TU/untag). Tissue expression pattern is summarized for all mRNA with log_2_ value >0.52 in the ‘Expression’ column, and the evidence for the tissue expression is referenced in the ‘Source’ column. BDGP = Berkeley Drosophila Genome Project *in situ* hybridization database. Additional file 6:
**Neural-specific mRNA 1-hour pulse expression levels and transcript half-lives.** Table listing normalized 1-hour pulse (t0) microarray signal intensity and normalized transcript half-life for neural-specific measurements. Values are the mean per transcript calculated by combining duplicate Pros > UPRT TU-Decay experiments. Standard deviation (SD) is listed for the 1-hour pulse and half-life values. Additional file 7:
**Neural versus whole embryo half-lives.** Table listing neural-specific and whole embryo half-lives and the ratio of neural/whole embryo half-life. The statistical significance of half-life differences was assessed by SAM analysis, and the *q* value (false discovery rate) is listed for each transcript (ns = not significant). Additional file 8:
**Half-life of neural fate and localized mRNAs.** Table listing 1-hour pulse (t0) and half-life for the ‘neural fate’ and ‘localized’ categories of mRNAs. Transcripts that contain a PRE motif in the 3’ UTR are noted in the ‘Contains PRE’ column. Additional file 9:
**There is no correlation between 3’ UTR length and mRNA half-life.** Scatter plot comparing 3’ UTR length and mRNA half-life for whole embryo and neural-specific datasets. Additional file 10:
**Decreased Pumilio expression in Pumilio RNAi embryos.** Anti-Pumilio western blot of control and Pumilio RNAi embryos. Additional file 11:
**Pumilio RNAi** ***versus*** **control half-lives.** Table listing Pumilio RNAi half-lives (average per transcript across three replicate experiments) and control half-lives (average per transcript across two replicate experiments) and the ratio of Pumilio RNAi/control half-life. The statistical significance of half-life differences was assessed by SAM analysis and the *q* value (false discovery rate) is listed for each transcript (ns = not significant).

## Abbreviations

4sUd: 4-thiouridine
actD: actinomysin D
ARE: aU-rich element
BDGP: Berkeley drosophila genome project
CNS: central nervous system
FDR: false discovery rate
GFP: green fluorescent protein
miR: microRNA
NMD: nonsense-mediate decay
PRE: Pumilio recognition element
RBP: RNA-binding protein
RNAi: RNA-interference
SAM: significance analysis of microarrays
T.g.UPRT: Toxoplasma gondii uracil phosphoribosyltransferase
t_1/2_: half-life
TU: thiouracil
UTR: untranslated region
