# Supplementary material for: Dynamic regulation of mRNA decay during neural development
Source: Neural Dev. 2015 Apr 21;10:11. doi: 10.1186/s13064-015-0038-6 (PMC4413985; doi:10.1186/s13064-015-0038-6)
Supplement: Additional file 1: — Transcription arrest in actinomycin D treated embryos. TU-RNA blot for control and ActD-treated embryos. [file 13064_2015_38_MOESM1_ESM.pdf]

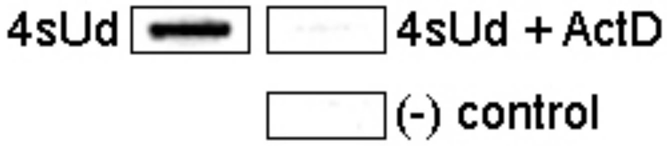


**Additional file 1**. Transcription inhibition in actinomycin D treated embryos. Permeabilized embryos were incubated in TU-tagging media containing 4-thiouridine (4sUd), 4-thiouridine + actinomycin D (4sUd + ActD), or media alone ((-) control). After two hours, total RNA was collected, biotinylated and analyzed by streptavidin-HRP slot-blot, using previously described methods for detecting TU-tagged RNA.
