# Supplementary material for: Dynamic regulation of mRNA decay during neural development
Source: Neural Dev. 2015 Apr 21;10:11. doi: 10.1186/s13064-015-0038-6 (PMC4413985; doi:10.1186/s13064-015-0038-6)
Supplement: Additional file 2: — Uracil number and transcript abundance do not bias mRNA decay measurements. Scatter plots of uracil number versus mRNA half-life (A) and transcript abundance in the 1-hour pulse sample versus mRNA half-life (B). [file 13064_2015_38_MOESM2_ESM.pdf]

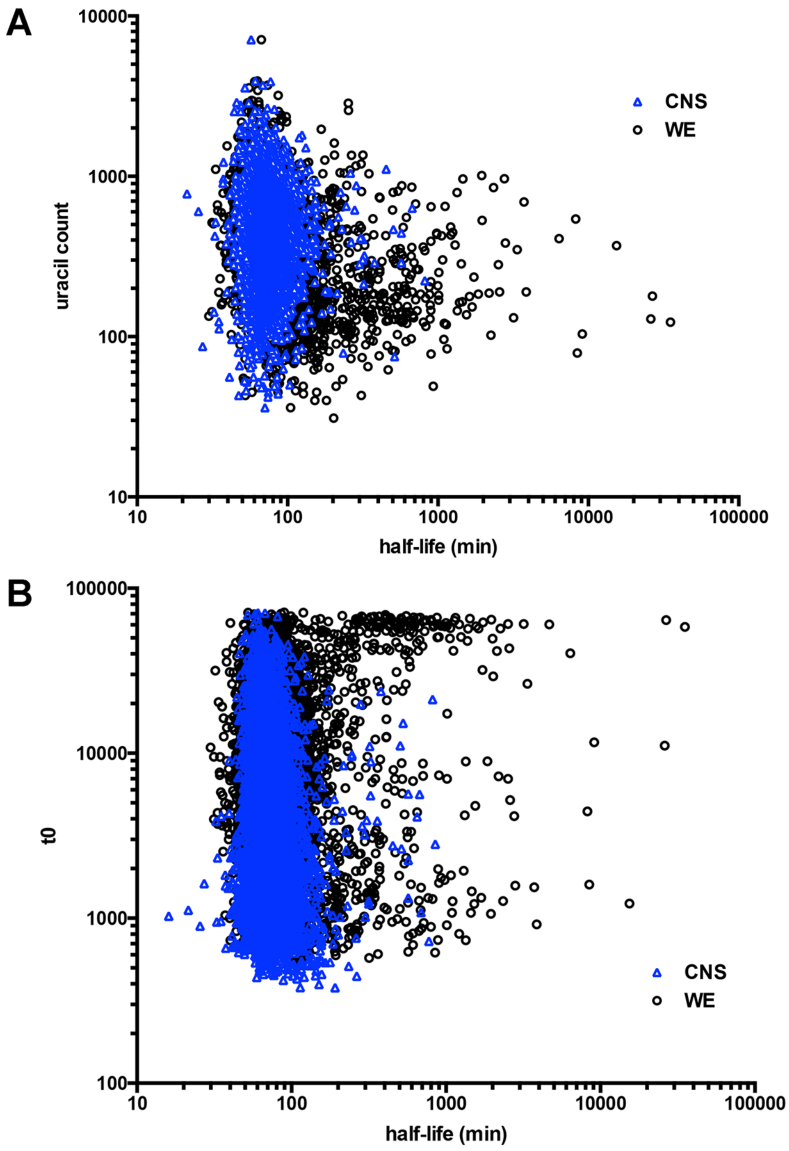

Additional file 2. Tests for potential biases in mRNA decay measurements. A. The number of uridines in a transcript does not influence decay measurements. Genome-wide mRNA half-lives obtained by whole embryo analysis (WE) and neural-specific analysis (CNS) are plotted on the x-axis and the number of uridines per transcript is plotted on the y-axis. All half-life values are included, regardless of goodness of fit to the exponential decay model (R^2 value). There was no significant correlation between uridine number and half-life in either dataset. B. Transcript abundance at the end of the 1-hour pulse does not influence decay measurements. Genome-wide mRNA half-lives are plotted on the x-axis and mRNA abundance at the end of the 1-hour pulse (t0 = chase time 0-hour) is plotted on the y-axis (signal of 65,000 is the maximum for the microarray). All half-life values are included, regardless of goodness of fit to the exponential decay model (R^2 value). There was no significant correlation between pre-chase transcript abundance and half-life in either dataset.
