# Supplementary material for: Dynamic regulation of mRNA decay during neural development
Source: Neural Dev. 2015 Apr 21;10:11. doi: 10.1186/s13064-015-0038-6 (PMC4413985; doi:10.1186/s13064-015-0038-6)
Supplement: Additional file 3: — Gene ontology categories of low-stability and high-stability mRNAs from UO and U + actD experiments. Table listing the gene ontology category enrichment for the 1,000 least stable and 1,000 most stable mRNAs in each dataset. [file 13064_2015_38_MOESM3_ESM.pdf]

Uridine only chase: High stability mRNAs					
GO term	Definition	P-value	FDR	Count	Fold Enrichment
0022626	Cytosolic ribosome	$1.5x10^{-49}$	$5.5x10^{-47}$	66	8.2
0022900	Electron transport chain	$1.8x10^{-9}$	$3.7x10^{-7}$	27	3.9
0031497	Chromatin assembly	$1.8x10^{-7}$	$1.9x10^{-5}$	15	5.4
0048024	Regulation of nuclear mRNA splicing	$3.4x10^{-6}$	$2.7x10^{-4}$	18	3.7
0010604	Macromolecule metabolic process	$7.3x10^{-5}$	$4.2x10^{-3}$	26	2.4

Uridine + ActD chase: High stability mRNAs					
GO term	Definition	P-value	FDR	Count	Fold Enrichment
0022626	Cytosolic ribosome	$1.1x10^{-49}$	$3.7x10^{-48}$	67	8.0
0022900	Electron transport chain	$1.1x10^{-17}$	$3.0x10^{-15}$	36	5.5
0005761	Mitochondrial ribosome	$2.1x10^{-5}$	$2.7x10^{-4}$	20	3.0
0048024	Regulation of nuclear mRNA splicing	$1.3x10^{-4}$	$8.0x10^{-3}$	15	3.3
0031497	Chromatin assembly	$1.7x10^{-4}$	$9.3x10^{-3}$	11	4.2

Uridine only chase: Low stability mRNAs					
GO term	Definition	P-value	FDR	Count	Fold Enrichment
0030182	Neuron differentiation	$2.1x10^{-11}$	$1.6x10^{-8}$	82	2.1
0007444	Imaginal disc development	$4.8x10^{-10}$	$1.2x10^{-7}$	79	2.1
0004672	Protein kinase activity	$6.9x10^{-7}$	$5.7x10^{-4}$	53	2.0
0014902	Myotube differentiation	$2.1x10^{-6}$	$1.1x10^{-4}$	12	5.6
0006350	Transcription	$1.2x10^{-4}$	$3.4x10^{-3}$	68	1.6

Uridine + ActD chase: Low stability mRNAs					
GO term	Definition	P-value	FDR	Count	Fold Enrichment
0007444	Imaginal disc development	$6.1x10^{-19}$	$1.4x10^{-15}$	99	2.5
0030182	Neuron differentiation	$1.2x10^{-17}$	$9.4x10^{-15}$	96	2.5
0004672	Protein kinase activity	$4.3x10^{-13}$	$1.1x10^{-10}$	67	2.6
0006350	Transcription	$1.2x10^{-9}$	$2.8x10^{-6}$	85	2.0
0030029	Actin based process	$2.8x10^{-9}$	$1.6x10^{-7}$	39	2.9

Additional file 3. Reproducible GO enrichment between uridine only and uridine + actinomycin D chase experiments. The top five most significant gene ontology categories enriched among the 1000 most stable transcripts (high stability mRNAs) and 1000 least stable transcripts (low stability mRNAs) for uridine chase and uridine + actinomycin D chase conditions. High stability results for each condition are listed first, followed by low stability results. FDR is the Benjamini-Hochberg false discovery rate.
