# Supplementary material for: Dynamic regulation of mRNA decay during neural development
Source: Neural Dev. 2015 Apr 21;10:11. doi: 10.1186/s13064-015-0038-6 (PMC4413985; doi:10.1186/s13064-015-0038-6)
Supplement: Additional file 9: — There is no correlation between 3’ UTR length and mRNA half-life. Scatter plot comparing 3’ UTR length and mRNA half-life for whole embryo and neural-specific datasets. [file 13064_2015_38_MOESM9_ESM.pdf]

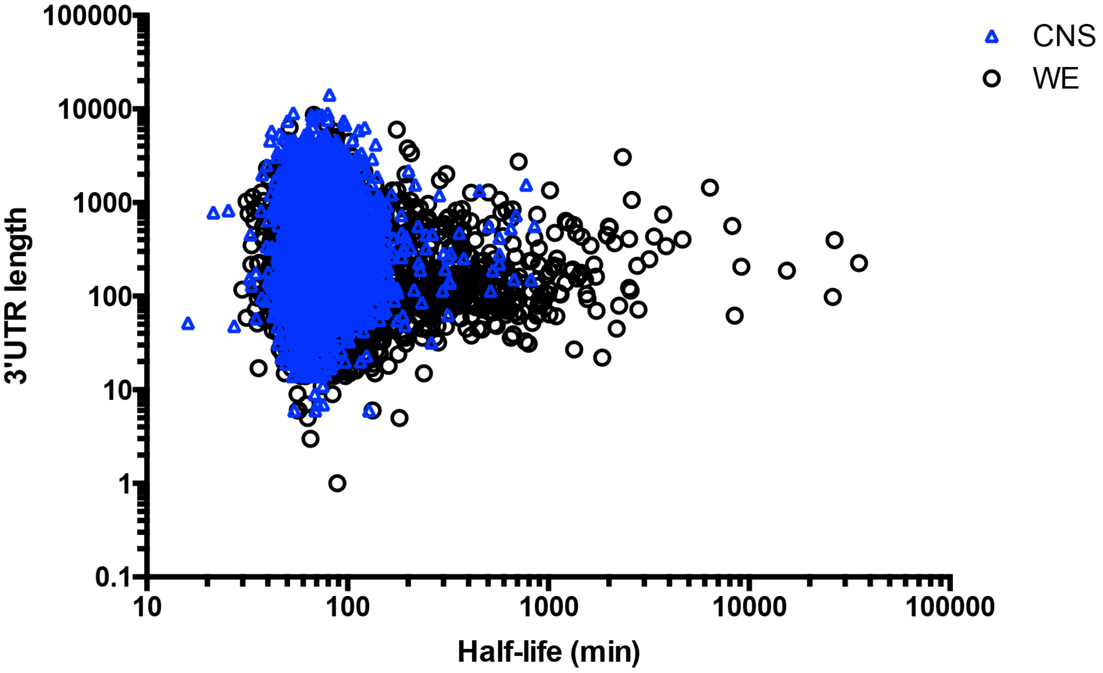

Additional file 9. There is no correlation between 3’ UTR length and mRNA half-life. Genome-wide mRNA half-lives obtained by whole embryo analysis (WE) and neural-specific analysis (CNS) are plotted on the x-axis and 3’ UTR length in nucleotides is plotted on the y-axis. All half-life values are included, regardless of goodness of fit to the exponential decay model (R^2 value). There was no significant correlation between 3’ UTR length and half-life in either dataset.
