# Supplementary material for: Dynamic regulation of mRNA decay during neural development
Source: Neural Dev. 2015 Apr 21;10:11. doi: 10.1186/s13064-015-0038-6 (PMC4413985; doi:10.1186/s13064-015-0038-6)
Supplement: Additional file 10: — Decreased Pumilio expression in Pumilio RNAi embryos. Anti-Pumilio western blot of control and Pumilio RNAi embryos. [file 13064_2015_38_MOESM10_ESM.pdf]

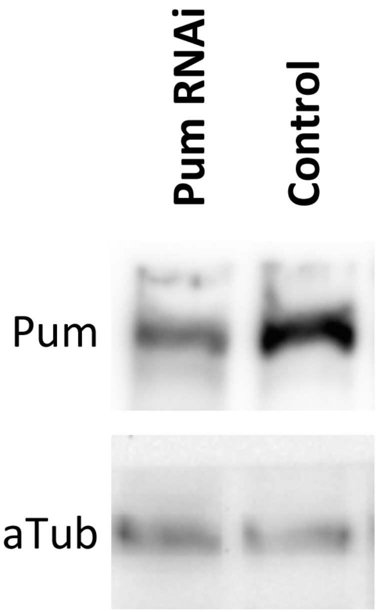

Supplemental Figure 4. Decreased Pumilio expression in *pumilio* RNAi embryos. Stage 12-15 Ubiquitious-Gal4 > UAS-Pum{RNAi} embryos were used for protein extraction and western blot against Pumilio and αTubulin. The Ubiquitous-Ga4 line was the GMR45H06 stock from the Fly Light Collection (BDSC stock number 45324).

Western blot method:
Embryos were dechorinated in bleach for 3 minutes, washed with PBS and homogenized in protein extraction buffer [50mM HEPES (pH 7.5), 150mM NaCl, 10% glycerol, 1% Triton X-100, 1.5mM MgCl, 1mM EGTA, 1 mini tab cOmplete protease inhibitor cocktail (Roche)]. Homogenate was cleared by centrifugation three times, quantified by Bradford Protein Assay (Thermo) and run in protein loading buffer [1X LDS, 100mM DTT] on a 4-20% Tris-Glycine polyacrylamide gel (Thermo) and transferred to a Hybond nitrocellulose membrane (GE Healthcare Life Sciences) according to manufacturers recommendations. Membranes were washed in TBST buffer [50mM Tris HCL (pH 7.5), 150 mM NaCl, 0.05% TWEEN-20] and blocked in 5% milk in TBST. Primary rat anti-Pum (1:200) and rat anti-αTubulin (1:300) were applied in blocking buffer overnight at 4°C. Secondary HRP conjugated anti-rat secondary antibodies were applied at 1:5000 (Pumilio) and 1:1000 (αTubulin) for 2 hours at room temperature. Blots were developed using SuperSignal West Pico Chemiluminescent Substrate (Thermo) for 5 minutes and imaged on a ChemiDoc MP System (BioRad).
